# Efficacy and safety of laparoscopic common bile duct exploration with primary closure and intraoperative endoscopic nasobiliary drainage for choledocholithiasis combined with cholecystolithiasis

**DOI:** 10.1007/s00464-022-09601-3

**Published:** 2022-10-07

**Authors:** Zhihong Zhang, Guohui Shao, Yanyang Li, Kejia Li, Guang Zhai, Xueyuan Dang, Zhitang Guo, Zhitian Shi, Renchao Zou, Lixin Liu, Hong Zhu, Bo Tang, Dong Wei, Lin Wang, Jiayun Ge

**Affiliations:** Department of Hepatobiliary and Pancreatic Surgery, Second Affiliated Hospital, Kunming Medical University, NO.374, Dianmian Rd., Wuhua District, Kunming, 650101 Yunnan China

**Keywords:** Cholecystolithiasis, Choledocholithiasis, Primary closure, Intraoperative endoscopic nasobiliary drainage (IO-ENBD), Antegrade

## Abstract

**Background:**

The need for intraoperative endoscopic nasobiliary drainage during laparoscopic cholecystectomy and laparoscopic common bile duct exploration with primary closure is controversial in the treatment of cholecystolithiasis combined with choledocholithiasis. The aim of this study was to evaluate the safety and efficacy of laparoscopic cholecystectomy + laparoscopic common bile duct exploration + intraoperative endoscopic nasobiliary drainage + primary closure (LC + LCBDE + IO-ENBD + PC). The safety of different intubation methods in IO-ENBD was also evaluated.

**Method:**

From January 2018 to January 2022, 168 consecutive patients with cholecystolithiasis combined with choledocholithiasis underwent surgical treatment in our institution. Patients were divided into two groups: group A (*n* = 96) underwent LC + LCBDE + IO-ENBD + PC and group B (*n* = 72) underwent LC + LCBDE + PC. Patient characteristics, perioperative indicators, complications, stone residual, and recurrence rates were analyzed. Group A was divided into two subgroups. In group A_1_, the nasobiliary drainage tube was placed in an anterograde way, and in group A_2_, nasobiliary drainage tube was placed in an anterograde–retrograde way. Perioperative indicators and complications were analyzed between subgroups.

**Results:**

No mortality in the two groups. The operation success rates in groups A and B were 97.9% (94/96) and 100% (72/72), respectively. In group A, two patients were converted to T-tube drainage. The stone clearance rates of group A and group B were 100% (96/96) and 98.6% (71/72), respectively. Common bile duct diameter was smaller in group A [10 vs. 12 mm, *P*** < **0.001] in baseline data. In perioperative indicators, group A had a longer operation time [165 vs.135 min, *P*** < **0.001], but group A had a shorter hospitalization time [10 vs.13 days, *P* = 0.002]. The overall complications were 7.3% (7/96) in group A and 12.5% (9/72) in group B. Postoperative bile leakage was less in group A [0% (0/96) vs. 5.6% (4/72), *P* = 0.032)]. There were no residual and recurrent stones in group A. And there were one residual stone and one recurrent stone in group B (all 1.4%). The median follow-up time was 12 months in group A and 6 months in group B. During the follow-up period, 2 (2.8%) patients in group B had a mild biliary stricture. At subgroup analysis, group A_1_ had shorter operation time [150 vs. 182.5 min, *P* < 0.001], shorter hospitalization time [9 vs. 10 days, *P* = 0.002], and fewer patients with postoperative elevated pancreatic enzymes [32.6% (15/46) vs. 68% (34/50), *P* = 0.001].

**Conclusion:**

LC + LCBDE + IO-ENBD + PC is safer and more effective than LC + LCBDE + PC because it reduces hospitalization time and avoids postoperative bile leakage. In the IO-ENBD procedure, the antegrade placement of the nasobiliary drainage tube is more feasible and effective because it reduces the operation time and hospitalization time, and also reduces injury to the duodenal papilla.

Depending on the presence or absence of symptoms, 5–20% of gallbladder stones were associated with common bile duct stones [1, 2]. The common bile duct stones can cause obstructive jaundice, cholangitis and acute pancreatitis, and may cause acute obstructive suppurative cholangitis in severe cases. Laparoscopic cholecystectomy is undoubtedly the standard treatment for cholecystolithiasis. Because of the popularity of various surgical equipment, a combined surgical approach using multiple endoscopes is constantly practiced. At the same time, combined treatment with multiple techniques to provide personalized and minimally invasive treatment for patients with biliary stones has also become one of the surgeons’ goals [3]. With the popularization of laparoscopy, choledochoscope and duodenoscope, the operation methods of common bile duct stones have been developed. At present, Laparoscopic cholecystectomy + endoscopic retrograde cholangiopancreatography and Laparoscopic cholecystectomy + laparoscopic common bile duct exploration are widely used in the treatment of cholecystolithiasis combined with choledocholithiasis.

Laparoscopic cholecystectomy + endoscopic retrograde cholangiopancreatography is usually performed in patients with small bile duct diameters. The advantage of endoscopic retrograde cholangiopancreatography is that it has no damage to common bile duct and avoids postoperative biliary stricture by taking stones through a physiological lumen. However, endoscopic retrograde cholangiopancreatography and endoscopic sphincterotomy may lead to pancreatitis, bleeding, perforation, and other complications [4], with total complications of 6–12% [5, 6]. In addition, endoscopic procedures such as endoscopic retrograde cholangiopancreatography and endoscopic sphincterotomy cannot avoid the injury of the duodenal papilla [7]. During Laparoscopic cholecystectomy + laparoscopic common bile duct exploration operation, the stone clearance rate is higher due to stone removal under direct vision through choledochoscope [5], and the operation success rate is also higher than Laparoscopic cholecystectomy + endoscopic retrograde cholangiopancreatography [8]. Meanwhile, the incidence of biliary stricture and bile leakage after laparoscopic common bile duct exploration significantly reduced in patients with bile duct dilatation [9]. T-tube drainage in laparoscopic common bile duct exploration can drain bile, support common bile duct, and facilitate the re-clearance of stones. Still, it will lead to T-tube-related complications such as bile leakage, body fluid and electrolyte loss and accidental T-tube detachment [10–12]. Subsequently, two meta-analyses evaluated the safety and effectiveness of Laparoscopic cholecystectomy + laparoscopic common bile duct exploration + primary closure and considered conventional placement of T-tubes was unreasonable [11, 13]. Another study confirmed the efficacy of this procedure and suggested that primary closure is also safe in patients with cholangitis [14]. However, it is worrying that the incidence of bile leakage after laparoscopic common bile duct exploration + primary closure remains 3–11.3% [14–18]. To replace the T-tube, and attempt to reduce complications, some institutions performed antegrade stent placement intraoperatively in laparoscopic common bile duct exploration. Regarding the effect of antegrade stent placement on bile leakage, the results of the two studies were not consistent [19, 20]. Similarly, the patients all required another gastrointestinal endoscope within a few weeks postoperatively to remove the stent.

To reduce the risk of postoperative bile leakage and not increase the complications associated with drainage tubes, some institutions have performed Laparoscopic cholecystectomy + laparoscopic common bile duct exploration + intraoperative endoscopic nasobiliary drainage + primary closure (LC + LCBDE + IO-ENBD + PC) [8, 21, 22]. However, few studies compare the efficacy of Laparoscopic cholecystectomy + laparoscopic common bile duct exploration + primary closure (LC + LCBDE + PC) and LC + LCBDE + IO-ENBD + PC and the need for IO-ENBD remains controversial due to the need for endoscopic equipment, technology and additional costs. This study evaluated the safety and efficacy of LC + LCBDE + IO-ENBD + PC, and we assessed the safety of two different IO-ENBD approaches, anterograde and anterograde–retrograde.

## Methods

### Patients

Patients included in this study were treated in our hospital from January 2018 to January 2022. Inclusion criteria were as follows: (1) diagnosis of cholecystolithiasis combined with choledocholithiasis; (2) written informed consent of the patient and completion of the procedure. The exclusion criteria were as follows:(1) history of cholecystectomy; (2) intrahepatic bile duct stones; (3) suspected gallbladder or bile duct tumors, or postoperative pathological diagnosis of gallbladder cancer; (4) laparotomy; (5) exploration through the cystic duct; (6) planned placement of a T-tube or placement of a biliary stent; (7) intraoperative cholangiography, including endoscopic retrograde cholangiopancreatography and cholangiography through nasobiliary drainage tube; (8) multi-stage surgery. With the approval of the Ethics Committee of the Second Affiliated Hospital of Kunming Medical University (NO. shen-PJ-2021-211), we retrospectively collected and analyzed the data of 168 patients. And the patients were divided into two groups: group A (*n* = 96) underwent LC + LCBDE + IO-ENBD + PC, group B (*n* = 70) underwent LC + LCBDE + PC. According to different IO-ENBD intubation methods, group A was divided into two subgroups. The patients of Group A_1_ performed IO-ENBD in an anterograde way, and the patients of group A_2_ performed IO-ENBD in an anterograde–retrograde way.

### Preoperative evaluation

Patients in both groups underwent blood test, abdominal ultrasound, magnetic resonance imaging, and magnetic resonance cholangiopancreatography. If necessary, an upper abdominal CT was performed to determine the condition of biliary pancreatitis. Some patients received percutaneous transhepatic gallbladder drainage or percutaneous transhepatic biliary drainage because of severe inflammation and jaundice.

### Surgical procedure

**Fig. 1:**
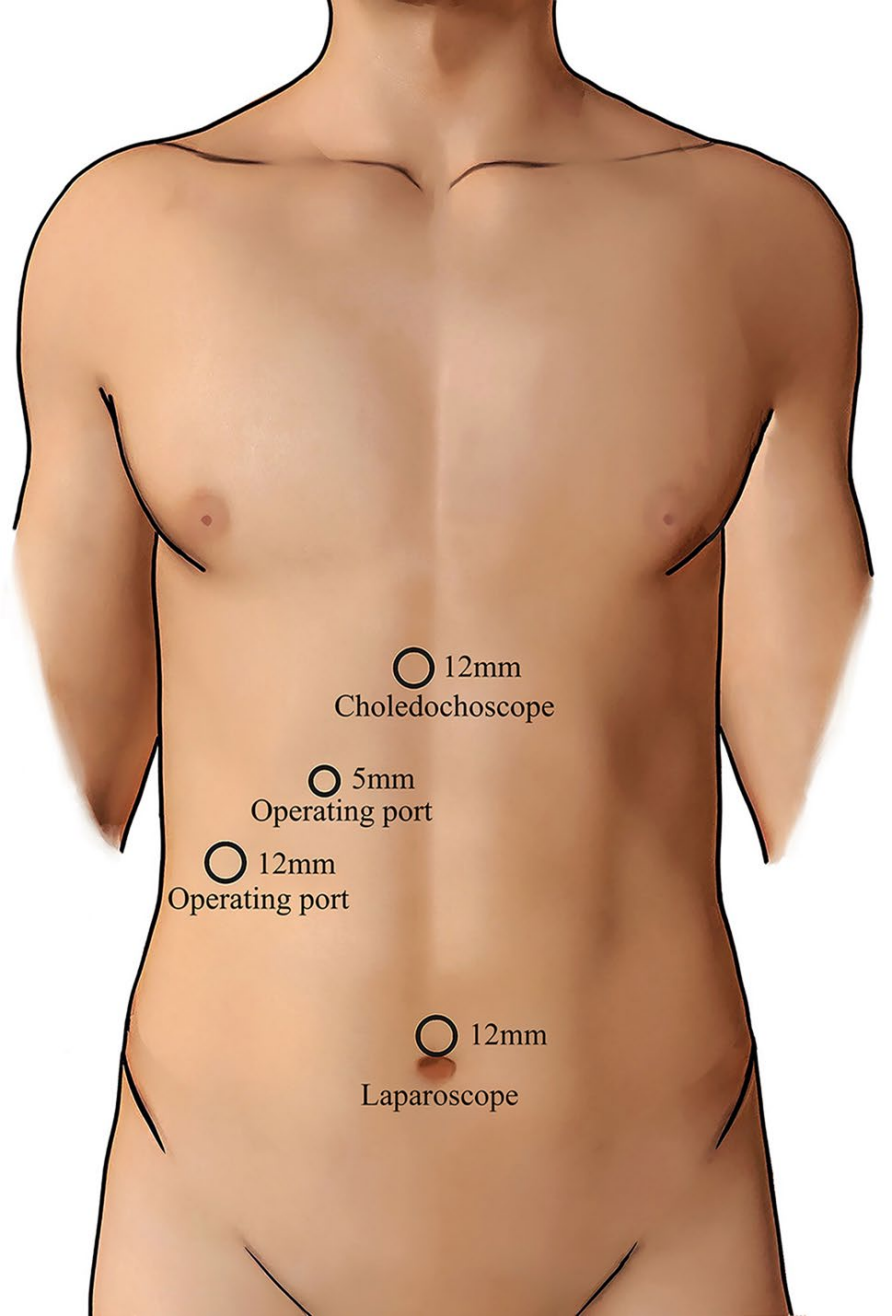
Schematic diagram showing the port sites of surgery

Three Trocar were placed in the patient’s upper abdomen, and another 5 mm Trocar was placed after the gallbladder was removed (Fig. 1). After common bile duct was cut longitudinally with a surgical knife blade or scissors, a choledochoscope was used to examine common bile duct and common hepatic duct, and a basket was used to remove stones.

**Fig. 2:**
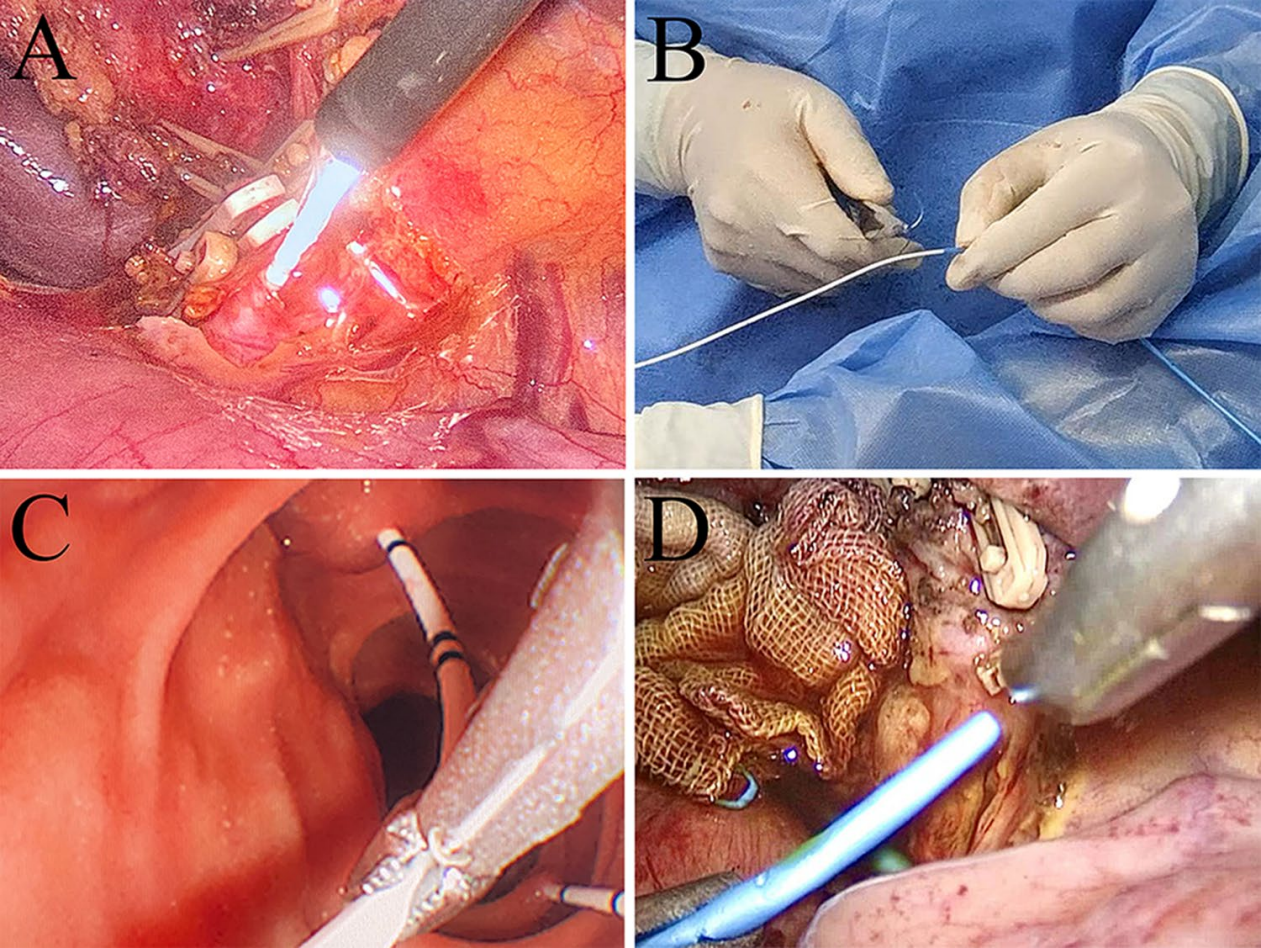
IO-ENBD procedure of group A_1_. **A** A 4F ureteral catheter was intubated anterogradely into the common bile duct using the cholangioscope and reached the duodenal papilla. **B** The ureteral catheter and nasobiliary drainage tube were connected by suture. **C** The ureteral catheter was clamped with an endoscope grasping forceps. **D** The nasobiliary drainage tube was inserted upward into the left or right hepatic duct

The IO-ENBD procedure in group A_1_ was as follows: First, a 4F ureteral catheter was intubated anterogradely into the common bile duct using the cholangioscope and reached the duodenal papilla (Fig. 2A). Then, the choledochoscope was removed. The other end of the ureteral catheter was inserted at the end of the nasobiliary drainage tube, with an insertion length of about 2–3 cm. The ureteral catheter and nasobiliary drainage tube were connected by suture (Fig. 2B). Subsequently, the ureteral catheter was found by a duodenoscope. And the ureteral catheter was clamped with an endoscope grasping forceps (Fig. 2C) and led out via the oral route. The nasobiliary drainage tube was constantly pulled out of the mouth by an assistant until the other side of the tube completely entered the trocar hole. Then, the tube was placed into the left or right hepatic duct (Fig. 2D). After that, the ureteral catheter was cut off completely and the nasobiliary drainage tube was led out via the oral-nasal route.

In group A_2_, the zebra guide wire was intubated anterogradely into the common bile duct using the cholangioscope and reached the duodenal papilla, and then the guide wire was found by the duodenoscope. The guide wire was pulled out of the mouth by endoscope grasping forceps, and then a nasobiliary drainage tube was placed by retrograde intubation under the guide of the wire. Intraoperative contrast procedures were not performed in Group A_2_, and other procedural steps were described in previous studies at our hospital [21].

After the nasobiliary drainage tube placement was completed, the assistant determined whether the tube was unobstructed by injecting sterile saline. All patients received continuous sutures of common bile duct using 3–0 or 4–0 absorbable barbed suture. Sterile saline was injected during suturing to flush out the blood clot. After the suture was completed, the assistant injected sterile saline again to determine whether there was bile leakage, and strengthened the suture. Abdominal drainage tubes were placed and antibiotics were administered intravenously within 24–48 h after surgery. The nasobiliary drainage tube was interruptedly clamped after 24 h, completely clamped after two days, and removed after three to seven days.

### Observation index and standard

The criteria for patient characteristics and perioperative indicators were as follows. The number of stones and diameter of CBD were obtained by preoperative ultrasound or magnetic resonance cholangiopancreatography. Duodenal diverticulum was diagnosed by preoperative imaging or intraoperative duodenoscopy. The definition and grading of postoperative bile leakage were based on the International Study Group of Liver Surgery criteria [23]. Post-ENBD pancreatitis was defined and graded according to the European Society of Gastrointestinal Endoscopy criteria [24]. Serum pancreatic enzymes were routinely measured 8, 12 and 24 h postoperatively. The biliary stricture was defined as postoperative magnetic resonance cholangiopancreatography indicating a narrowing of common bile duct at the suture. The effect of IO-ENBD on the duodenal papilla was reflected by post-ENBD pancreatitis and postoperative pancreatic enzyme elevation when comparing the subgroups. Postoperative pancreatic enzyme elevation was any postoperative increase in serum amylase or lipase above normal.

### Data analysis

Measurement data were expressed as $\overline{x}\, \pm \,s$ or [*M* (*P*_*25*_, *P*_*75*_)], and group comparison was performed using the Student t-test or Mann–Whitney U test. The count data were expressed as rates, and group comparison was performed using the Pearson Chi-Square test or Fisher exact test. *P* < 0.05 was considered statistically significant. Statistical analysis was performed using SPSS 26.0.

## Results

### Patient characteristics

The patient characteristics results are shown in Table 1. There were no significant differences between group A and group B in most of indexes (*P* > 0.05). However, the diameter of common bile duct in group A was significantly smaller than that in group B [10 (9, 11) mm vs. 12 (10, 15) mm, (*P*** < **0.001)].

**Table 1:** Characteristics of patients ($\overline{x}\, \pm \,s$), *M* (*P*_*25*_*, P*_*75*_) or [n (%)]

Characteristics	Group A (*n* = 96)	Group B (*n* = 72)	*P*-value
Age (year)	55.5 ± 16.0	57.9 ± 17.6	0.357
Sex			
Male	42 (43.8)	29 (40.3)	0.652
Female	54 (56.3)	43 (59.7)	0.652
Presentations			
Jaundice	18 (18.8)	21 (29.2)	0.114
History			
Biliary colic	85 (88.5)	65 (90.3)	0.719
Cholangitis	10 (10.4)	15 (20.8)	0.060
Biliary pancreatitis	13 (13.5)	7 (9.7)	0.449
PTCD or PTGD	10 (10.4)	13 (18.1)	0.154
Multiple stones in CBD	58 (60.4)	39 (54.2)	0.417
Diameter of CBD (mm)	10 (9, 11)	12 (10, 15)	< 0.001
Duodenal diverticulum	3 (3.1)	4 (5.6)	0.463

*CBD* common bile duct; *PTCD* percutaneous transhepatic biliary drainage; *PTGD* percutaneous transhepatic gallbladder drainage

### Perioperative results

Perioperative results are presented in Table 2. No patients in either group were converted to open surgery. The success rate was 97.9% (94/96) in group A and 100% (72/72) in group B. One case in group A_1_ was converted to T-tube drainage because duodenoscopy was difficult to pass through the pylorus, and one case in group A_2_ was converted to T-tube drainage because duodenal diverticulum prevented endoscopic retrograde intubation. The operative time in group A was significantly longer than that in group B (*P*** < **0.001). The hospitalization time in group A was shorter than that in group B (*P*** = **0.002). The Hospitalization expenses in group A were slightly higher than that in group B, but there was no significant difference between the two groups (*P* > 0.05). The time of abdominal drainage in group A was significantly shorter than that in group B [3 (2, 4) days vs. 5 (4, 7) days, (*P* < 0.001)].

**Table 2:** Comparison of perioperative indexes *M* (*P*_*25*_*,P*_*75*_)

Outcomes	Group A (*n* = 96)	Group B (*n* = 72)	*P*-value
Operation time (min)	165 (145, 204)	135 (115, 152.8)	< 0.001
Intraoperative bleeding (ml)	30 (16.3, 50)	30 (10, 50)	0.610
Hospitalization time (day)	10 (8, 12)	13 (8, 18)	0.002
Hospitalization expenses (yuan)	29,655 (25,773.5, 31,443)	27,165 (23,340.3, 32,805)	0.151
Abdominal drainage time (day)	3 (2, 4)	5 (4, 7)	< 0.001

### Postoperative complications

The complications are shown in Table 3. There were no significant differences in most of the complications between the two groups (*P* > 0.05). However, 4 patients (5.6%) in group B had postoperative bile leakage, and there was no bile leakage in group A (*P* = 0.032).The classification and management results of complications are shown in Table 4. Complications were classified according to the Clavien–Dindo classification system [25]. Bile leakage was graded according to the International Study Group of Liver Surgery criteria [23]. In group B, bile peritonitis was found in 1 case due to grade C bile leakage, and residual stones were found. The stones were removed by laparoscopic common bile duct exploration again, and the bile leakage stopped after T tube drainage. However, the patient returned to the hospital 3 months after the operation for T-tube angiography showed asymptomatic mild biliary stricture. During the follow-up period, two patients with mild biliary stricture had no symptoms.

**Table 3:** Comparison of postoperative indexes [n (%)]

Outcomes	Group A (*n* = 96)	Group B (*n* = 72)	*P*-value
Overall complications	7 (7.3)	9 (12.5)	0.255
Pulmonary infection	2 (2.1)	1 (1.4)	1.000
Biliary tract infection	0	2 (2.8)	0.182
Bile leakage	0	4 (5.6)	0.032
PEP	5 (5.2)	0	0.072
Biliary stricture	0	2 (2.8)	0.182
Residual stone	0	1 (1.4)	0.429
Stone recurrence	0	1 (1.4)	0.429

*PEP* post-ENBD pancreatitis

**Table 4:** Complications and management [n (%)]

Grade^a^	Complications	Group A	Group B	Management
I	Bile leakage	–	2	Extension of abdominal drainage
	Biliary stricture	–	2	Follow-up and observation
II	Biliary tract infection	–	2	Intravenous antibiotics
	Pulmonary infection	2	1	Intravenous antibiotics
	PEP	5	–	Intravenous somatostatin and antibiotics
IIIa	Bile leakage	–	1	ERCP and ENBD under local anesthesia
IIIb	Bile leakage	–	1	LCBDE + T tube drainage under general anesthesia

^a^Clavien–Dindo classification of surgical complications. No IVa, IVb, V complications. *ERCP* endoscopic retrograde cholangiopancreatography

### Stone clearance results

The stone clearance and recurrence rates are shown in Table 3. Abdominal ultrasound was used in patients with persistent abdominal pain and jaundice after the operation to identify residual stones. Some patients in group A underwent nasal cholangiography to identify residual stones. The stone clearance rates of group A and group B were 100% (96/96) and 98.6% (71/72), respectively (*P* > 0.05). In group B, there was 1 case with residual stones, which had persistent jaundice and bile leakage, leading to bile peritonitis. LC + LCBDE + T tube drainage was performed again one week after the operation.

### Subgroup comparison results

The results of the subgroup comparison are shown in Table 5. The operation time in group A_1_ was significantly shorter than that in group A_2_ (*P* < 0.001). Hospitalization time in group A_1_ was also shorter than that in group A_2_ (*P* = 0.002). In addition, fewer patients in group A1 than in group A2 had elevated pancreatic enzymes (*P* = 0.001).

**Table 5:** Comparison of perioperative and postoperative indexes in subgroups *M* (*P*_*25*_*, P*_*75*_) or [n (%)]

Outcomes	Group A_1_ (n = 46)	Group A_2_ (n = 50)	*P*-value
Operation time (min)	150 (132.8, 177)	182.5 (165, 257.8)	< 0.001
Intraoperative bleeding (ml)	30 (10, 50)	50 (20, 80)	0.092
Hospitalization time (day)	9 (7,11)	10 (9, 13.5)	0.002
Hospitalization expenses (yuan)	28,521.5 (25,078, 30,500.3)	30,228 (26,483, 33,537.3)	0.085
Overall complications	2 (4.3)	5 (10)	0.438
PEP	1 (2.2)	4 (8)	0.364
Postoperative pancreatic enzyme elevation	15 (32.6)	34 (68)	0.001

### Follow-up results

The follow-up results are shown in Table 3. Follow-up visits were made through outpatient or telephone interviews. Follow-up was performed every 3 months within half a year after discharge and every 6 months thereafter. During telephone interviews, patients were asked about abdominal pain, fever, jaundice and other medical records. Blood cell and liver function tests were performed at outpatient follow-up, and magnetic resonance cholangiopancreatography was performed if necessary. All patients completed at least one follow-up visit by April 2022. Follow-up time was 12 (6, 18) (range: 3–48 months) in group A and 6 (6, 18) (range: 3–48 months) in group B. In 168 patients, the overall stone recurrence rate and biliary stricture rate were 0.60% and 1.2%, respectively. One case (1.4%) in group B was found to have stone recurrence 15 months after the operation, and then endoscopic retrograde cholangiopancreatography + endoscopic sphincterotomy was performed. In group B, 2 (2.8%) patients were found to have mild biliary stricture, but no treatment was required, and no symptoms remained during the follow-up period.

Patient experience data and scores for nasobiliary drain include the following. Throat symptoms: none (10 points), mild discomfort (6 points) or pain (3 points); gastrointestinal symptoms: none (10 points), mild nausea (6 points), or severe nausea leading to vomiting (3 points); accidental nasobiliary drainage tube detachment: no (10 points), yes (3 points); nasobiliary drainage time: ≤ 5 days (10points), > 5 days but ≤ 7 days (6 points), > 7 days (3 points). Patients with a total score ≥ 32(80%) points were considered to be well satisfied, ≥ 24 but < 32 points were considered to be generally satisfied, and < 24(60%) points were considered to be poorly satisfied. After excluding the 2 patients in group A who were converted to T-tube drainage, the results showed that 86/94(91.49%) patients were well satisfied with nasobiliary drainage, 8/94(8.51%) patients were generally satisfied. No patients were poorly satisfied.

### Discussion

This study found that patients with LC + LCBDE + PC had a larger diameter of bile duct; LC + LCBDE + IO-ENBD + PC significantly prolonged operation time, but avoided bile leakage and shortened hospital stay; the placement of nasobiliary drainage tube in an anterograde way significantly shortened operation time and hospitalization time compared with anterograde–retrograde way, and also reduced the impact on duodenal papilla.

Biliary stricture after laparoscopic common bile duct exploration is related to other factors such as suture technique and blood supply of the bile duct, so laparoscopic common bile duct exploration should be performed by skilled surgeons, and the indication should be strictly controlled. Several studies indicate that laparoscopic common bile duct exploration is safer in patients with common bile duct diameter ≥ 8 mm [9, 16]. In fact, in our hospital, to avoid biliary stricture after laparoscopic common bile duct exploration, we usually performed primary closure in patients with larger bile duct diameters, which resulted in a significant difference in common bile duct diameter between Group A and Group B. This may prove that IO-ENBD reduces the limitation of primary closure on the diameter of the biliary tract, and more patients with a diameter of common bile duct of about 10 mm can safely perform primary closure with simultaneous IO-ENBD, which is equivalent to IO-ENBD expanding the indication of primary closure. Although the diameter of common bile duct was larger, 2 (2.8%) patients in Group B had mild biliary strictures at follow-up, higher than those reported by Pei Yin and Nuria Estelle ´s Vidagany [8, 16]. These two patients did not require any invasive treatment and were asymptomatic during the follow-up period.

LC + LCBDE + IO-ENBD + PC prolonged the procedure due to the need for additional endoscopic procedures. In this study, the median operation time in group A was 165 min, significantly higher than in group B at 135 min (*P* < 0.001). These data are comparable to those reported by Jie Hua and Victor Vakayil [15, 26], but higher than Pei Yin’s [8]. Our colleagues previously reported LC + LCBDE + PC + intraoperative endoscopic retrograde cholangiopancreatography + IO-ENBD for specific patients [21], but our institution no longer routinely conducts intraoperative endoscopic retrograde cholangiopancreatography before IO-ENBD. This is because choledochoscopy can remove stones under direct vision, and we believe that intraoperative endoscopic retrograde cholangiopancreatography after primary closure is redundant in most cases. Furthermore, endoscopic retrograde cholangiopancreatography requires additional retrograde intubation procedures that significantly increase operation time and risk of complications, which is not consistent with the original intent of LC + LCBDE + IO-ENBD + PC.

Since LC + LCBDE + PC has no biliary drainage, bile leakage is one of the most worrying postoperative complications. Several studies have shown that removing the gallbladder as a pressure reservoir results in an increase in Oddi sphincter pressure [27], which leads to an increase in choledochal pressure [28]. This also explains the gradual increase in common bile duct diameter in some patients after cholecystectomy. Although bile leakage is closely related to the suture technique, we still believe that the rapid changes of Oddi sphincter dynamics and the increase of common bile duct pressure after cholecystectomy may be one of the causes of bile leakage after LC + LCBDE + PC. Based on these factors, IO-ENBD should be considered necessary in LC + LCBDE + PC because it can help overcome this period of rapid change in pressure. In group A, the operation time was significantly prolonged, but the postoperative bile leakage was avoided. In group B, there were 4 cases (5.6%) postoperative bile leakage. Among them, two patients with grade A bile leakage had prolonged abdominal drainage; 1 patient with grade B bile leakage underwent endoscopic retrograde cholangiopancreatography and ENBD under local anesthesia; and 1 patient with grade C bile leakage underwent laparoscopic common bile duct exploration and T-tube drainage under general anesthesia (Table 4). Biliary drainage can reduce the pressure of common bile duct and promote the healing of the suture site, but the drainage method, including T tube and stent, will bring many burdens. Nasobiliary drainage tube has the advantages of simple nursing, accurate drainage and early removal [8]. Usually, the tube is removed three to seven days after surgery, and no routine radiography is required before removal unless the patient has clinical symptoms. It is noteworthy that two patients in Group A failed IO-ENBD and were converted to T-tube drainage. One patient in group A_1_ had gastric pyloric stenosis due to a long-term ulcer, and duodenoscopy was difficult to pass; one patient in group A_2_ had retrograde intubation difficulty due to duodenal diverticulum. Physiological conditions of the stomach and duodenum restrict IO-ENBD to some extent, so the previous medical history and preoperative imaging examination should be paid more attention to, and T-tube drainage can be used as an alternative operation in case of IO-ENBD failure.

In terms of overall complications, there was no statistical difference between the two groups. But the Hospitalization time in group A was still shorter than that in group B (*P* = 0.002). The reason was that there was no bile duct drainage in group B, and the doctor tended to prolong the hospitalization time for observation. The results showed that the abdominal drainage time of group A was significantly shorter than that of group B, which also pointed out that group B tended to have a longer abdominal drainage time to observe whether there was bile leakage. In contrast, nasobiliary drainage tube was placed in group A, and early discharge was acceptable. Follow up results also showed that 91.49% of patients were satisfied with nasobiliary drainage. A small number of patients were discharged with nasobiliary drainage tubes, and these patients returned to the outpatient department one week after the operation to assess and remove the tubes. Several previous studies have also shown the advantages of LC + LCBDE + IO-ENBD + PC in reducing hospital stays [8, 22]. IO-ENBD is difficult to accept because it requires additional costs, including nasobiliary drainage tube and guidewire. There was no significant difference between group A and group B in hospitalization cost (*P* > 0.05) in this study. Compared with group A, the hospitalization time in group B was longer, which resulted in the hospitalization cost of group B being slightly higher. Hospitalization costs in this study are similar to those in another study [8]. Stone clearance and stone recurrence rates were similar between the two groups, and there was no statistical difference between the two groups (*P* > 0.05).

To increase intubation success and reduce postoperative complications, endoscopic retrograde cholangiopancreatography was standardized in the guidelines of the European Society for Gastrointestinal Endoscopy [29]. However, retrograde intubation damage to the duodenal papilla is challenging to avoid. The standard endoscopic retrograde cholangiopancreatography retrograde intubation procedure was not performed in Group A patients, but post-ENBD pancreatitis still occurred in 5 patients (5.2%). It is worth mentioning that all patients in Group A performed IO-ENBD in a manner that reduced duodenal papillary injury. Group A_1_ was treated with a completely anterograde method, and the drainage tube only passed through the duodenal papilla once. Group A_2_ needed to pass through the duodenal papilla twice. Group A_1_ completely avoided retrograde intubation, while group A_2_ was retrogradely placed with nasobiliary drainage tube guided by wire, which was still retrograde intubation. Compared with group A_2_, there were fewer patients in group A_1_ with postoperative elevated pancreatic enzymes (*P* = 0.001), demonstrating that the anterograde approach used in group A_1_ significantly reduced the effect on the duodenal papilla. The incidence of post-ENBD pancreatitis was not significantly different (*P* > 0.05). Other reports use the same approach as Group A_2_ [21, 22, 30]. To the best of our knowledge, IO-ENBD has not been reported using the full antegrade method. We refer to the concept of "antegrade" proposed in previous studies [19, 31]. Ureteral catheters were used in group A_1_ to guide the nasobiliary drainage tubes without the need for a guidewire and retrograde intubation procedures and were usually successful at one time. Subgroup analysis also showed that the operation time in group A_1_ was significantly shorter than that in group A_2_ (*P* < 0.001). Antegrade mode is more convenient, which can reduce the operation difficulty of IO-ENBD, shorten the learning curve, and be performed with gastroscopy, reducing the equipment requirements. In addition, the subgroup analysis showed that the A_1_ group had shorter hospital stays than the A_2_ group (*P* = 0.002). This was believed to be associated with elevated pancreatic enzymes after surgery, leading to longer postoperative observation times in group A_2_. Zebra guidewire was not required in group A_1_ and was cost-effective, although there was no statistically significant difference in hospitalization costs between subgroups (*P* > 0.05).

Retrospective analysis is a major limitation of this study, and the completion of procedures by different teams is one of the possible causes of bias. There are few studies on whether LC + LCBDE + PC needs IO-ENBD, and some studies have proposed that the size of common bile duct stone is related to the pressure of common bile duct [32]. Determining the indication of IO-ENBD according to the diameter of common bile duct and the size of the stone is one of the problems to be further solved. At the same time, this study reported for the first time on IO-ENBD performed in full compliance with the concept of “antegrade” and showed good results in the near term. But this is our single-center experience, and other studies are needed to confirm it. Another multicenter clinical study conducted by our team is also in progress. In addition, there was no statistical difference in the incidence of post-ENBD pancreatitis between the A_1_ and A_2_ groups in this study (*P* = 0.072), possibly due to the small sample size. A more extensive study is expected to verify the effect of different intubation methods on post-ENBD pancreatitis.

In summary, LC + LCBDE + PC + IO-ENBD is safer and more effective than LC + LCBDE + PC because it reduces hospitalization time and avoids postoperative bile leakage. At the same time, in the IO-ENBD procedure, the antegrade placement of the nasobiliary drainage tube is more feasible and effective because it reduces the operation time and hospitalization time, and also reduces injury to the duodenal papilla.
